# Prognostic Value of Inflammatory and Nutritional Indices in Patients Undergoing Permanent Pacemaker Implantation for Degenerative Complete Atrioventricular Block

**DOI:** 10.3390/medicina62091741

**Published:** 2026-09-10

**Authors:** Murat Erdem Alp, Cemal Ozanalp, Güngör İlayda Bostancı Alp, Merve Kertmen, Eyüp Özkan, Veli Polat, Süleyman Barutçu, Taylan Akgün

**Affiliations:** Department of Cardiology, Istanbul Başakşehir Çam and Sakura City Hospital, Istanbul 34480, Turkey; cemalozanalp@gmail.com (C.O.); gilaydabostanci@hotmail.com (G.İ.B.A.); drmervekertmen@gmail.com (M.K.); dreyupozkan@gmail.com (E.Ö.); dr.velipolat@gmail.com (V.P.); slybrtcu@gmail.com (S.B.); taylanakgun@gmail.com (T.A.)

**Keywords:** complete atrioventricular block, permanent pacemaker, prognostic nutritional index, geriatric nutritional risk index, inflammatory indices, nutritional status, all-cause mortality

## Abstract

*Background and Objectives*: Systemic inflammation and nutritional impairment may influence outcomes after permanent pacemaker implantation, but the prognostic value of composite indices in degenerative complete atrioventricular block remains unclear. We evaluated the associations of routinely available inflammatory and nutritional indices with long-term all-cause mortality in this population. *Materials and Methods*: This retrospective, single-center study included 272 patients who underwent permanent pacemaker implantation for isolated complete atrioventricular block attributed to degenerative conduction system disease between August 2020 and July 2024. Baseline laboratory values were used to calculate the prognostic nutritional index (PNI), geriatric nutritional risk index (GNRI), neutrophil-to-lymphocyte ratio, lymphocyte-to-monocyte ratio, systemic immune-inflammation index, systemic inflammation response index, and pan-immune-inflammation value. The primary endpoint was all-cause mortality during follow-up. Discriminatory performance was assessed using receiver operating characteristic (ROC) analysis, and independent associations were evaluated using multivariable Cox regression. *Results*: During a mean follow-up of 27.8 ± 16.6 months, 69 patients (25.4%) died. Compared with survivors, non-survivors were older and had lower hemoglobin, lymphocyte count, albumin, estimated glomerular filtration rate, GNRI, LMR, and PNI values, whereas C-reactive protein, NLR, and SIRI values were higher. PNI had the numerically highest AUC for mortality prediction (AUC: 0.720, 95% CI: 0.648–0.793; *p* < 0.001), corresponding to moderate discriminatory performance, whereas GNRI showed fair discrimination (AUC: 0.677, 95% CI: 0.589–0.766; *p* < 0.001). In multivariable Cox regression, PNI remained independently associated with mortality (HR: 0.945, 95% CI: 0.909–0.982; *p* = 0.004). *Conclusions*: Among the evaluated inflammatory and nutritional indices, PNI demonstrated the numerically highest discriminatory performance and remained independently associated with long-term all-cause mortality. PNI may therefore serve as a readily available adjunct to clinical risk stratification in patients undergoing permanent pacemaker implantation for degenerative complete atrioventricular block; however, external validation is required.

## 1. Introduction

The number of patients requiring permanent pacemaker implantation has steadily increased because of the growing prevalence of degenerative conduction system disease and expanding indications for cardiac pacing [1]. Contemporary primary-care data have shown that atrioventricular block is associated with reduced survival and that third-degree atrioventricular block carries the poorest prognosis among atrioventricular block subtypes [2].

Complete atrioventricular block requiring permanent pacemaker implantation may result from various causes, including medications, acute coronary syndromes, toxins, electrolyte disturbances, cardiac surgery, catheter-based interventions, and congenital conduction disorders [1]. In routine clinical practice, however, permanent pacemaker implantation is frequently required in patients with conduction system disease caused by degenerative processes [1]. This subgroup is clinically important because degenerative complete atrioventricular block commonly affects older adults with comorbidities and reduced physiological reserve [2,3,4,5,6].

Recent pacemaker cohorts have identified advanced age, diabetes mellitus, reduced left ventricular systolic function, anemia, renal dysfunction, structural heart disease, and frailty as adverse prognostic factors [3,5,6]. Serum albumin, a routinely available marker of nutritional status and systemic disease burden, has also been associated with mortality in this population [4]. However, serum albumin alone may not fully capture the interplay among nutritional reserve, immune competence, systemic inflammation, and frailty, all of which may influence long-term outcomes after pacemaker implantation [4,5,7,8].

In this context, composite inflammatory and nutritional indices derived from routinely available laboratory parameters have attracted increasing interest as prognostic markers across clinical settings [7,8,9,10,11,12,13,14,15,16,17,18,19,20]. The prognostic nutritional index combines serum albumin and lymphocyte count and therefore reflects both nutritional status and immune-inflammatory balance [7]. Similarly, the geriatric nutritional risk index was developed as a practical tool for assessing nutrition-related risk in older medical patients [8]. Although PNI and GNRI were originally developed in surgical oncology and geriatric medicine, respectively, subsequent evidence has supported their prognostic application in cardiovascular populations [9,10,16,17,19,20] and, for PNI, specifically in patients with cardiac pacemakers [9]. These indices do not require clinically overt malnutrition to provide prognostic information; instead, they may capture subclinical depletion of nutritional reserves, systemic inflammation, and reduced physiological resilience. This may be particularly relevant to degenerative complete atrioventricular block, which predominantly affects older adults with comorbidities and potential frailty. Other hematologic indices, including the neutrophil-to-lymphocyte ratio, lymphocyte-to-monocyte ratio, systemic immune-inflammation index, systemic inflammation response index, and pan-immune-inflammation value, may reflect different aspects of systemic inflammation and immune dysregulation [11,12,13,14,15,18]. These indices can be calculated from routinely obtained laboratory parameters, require no additional testing costs, and are readily available in routine clinical practice.

Nevertheless, the prognostic value of inflammatory and nutritional indices has not been well established in patients undergoing permanent pacemaker implantation for isolated complete atrioventricular block caused by degenerative conduction system disease. Previous pacemaker studies have generally included heterogeneous pacing indications and have not specifically examined this clinically uniform subgroup [3,4,5,6,9]. Therefore, the present study aimed to evaluate the associations of routinely available inflammatory and nutritional indices with long-term all-cause mortality in patients undergoing permanent pacemaker implantation for degenerative complete atrioventricular block and to determine whether these indices provide prognostic information beyond conventional clinical and laboratory parameters.

## 2. Materials and Methods

### 2.1. Study Population and Design

This retrospective, single-center, observational study included patients who underwent permanent pacemaker implantation for complete atrioventricular block between August 2020 and July 2024. Patients were eligible if they underwent permanent pacemaker implantation for isolated complete atrioventricular block attributed to degenerative conduction system disease.

The exclusion criteria were pacemaker implantation for conduction disorders other than complete atrioventricular block; atrioventricular block attributable to reversible, secondary, iatrogenic, or congenital causes; drug-induced atrioventricular block; acute coronary syndrome or acute myocardial infarction; electrolyte disturbances; toxin-related atrioventricular block; acute systemic illness; active infection; atrioventricular block following transcatheter aortic valve implantation or cardiac surgery; post-ablation atrioventricular block; congenital heart block; age < 18 years; and the presence of rheumatic, hematologic, malignant, chronic inflammatory, or autoimmune diseases.

The classification of degenerative conduction system disease was adjudicated retrospectively by the study investigators based on a review of institutional medical records. Degenerative disease was defined as isolated complete atrioventricular block requiring permanent pacemaker implantation after the exclusion of potentially reversible, secondary, iatrogenic, congenital, inflammatory, infectious, malignant, or hematologic causes.

Demographic characteristics, comorbidities, pacemaker type, and laboratory parameters were obtained from institutional medical records. Pacemaker type was categorized as single- or dual-chamber implantation and was retained only as an adjustment covariate to account for differences related to device selection; no causal comparison of pacing modes was intended. Coronary artery disease, diabetes mellitus, hypertension, and atrial fibrillation were recorded from the patients’ medical histories and available clinical documentation. Heart failure was defined in accordance with the universal definition as a clinical syndrome with current or previous symptoms and/or signs attributable to a structural or functional cardiac abnormality, corroborated by elevated natriuretic peptide concentrations and/or objective evidence of pulmonary or systemic congestion [21]. For this retrospective study, heart failure status was adjudicated from the treating physician’s documented diagnosis and the available clinical, laboratory, and echocardiographic records. Reduced left ventricular ejection fraction alone, in the absence of a documented clinical heart failure syndrome, was not considered sufficient.

#### Laboratory Measurements and Calculation of Inflammatory and Nutritional Indices

Baseline laboratory parameters were obtained from blood samples collected within 24 h before pacemaker implantation. These included hemoglobin, white blood cell count, neutrophil count, lymphocyte count, monocyte count, platelet count, serum creatinine, estimated glomerular filtration rate, serum albumin, total protein, C-reactive protein, electrolytes, and other routine biochemical parameters. The estimated glomerular filtration rate was calculated using the Chronic Kidney Disease Epidemiology Collaboration (CKD-EPI) creatinine equation [22].

Inflammatory and nutritional indices were calculated from baseline laboratory values according to previously described formulas. The prognostic nutritional index was calculated using the following formula [7]:

PNI = serum albumin (g/L) + 5 × lymphocyte count (10^9^/L)

The geriatric nutritional risk index was calculated as follows [8]:

GNRI = 1.489 × serum albumin (g/L) + 41.7 × (actual body weight/ideal body weight)

Ideal body weight was calculated as 22 × height (m)^2^, corresponding to a reference BMI of 22 kg/m^2^. The neutrophil-to-lymphocyte ratio, lymphocyte-to-monocyte ratio, systemic immune-inflammation index, systemic inflammation response index, and pan-immune-inflammation value were calculated using the following formulas [11,12,13,14,15]:

NLR = neutrophil count/lymphocyte count

LMR = lymphocyte count/monocyte count

SII = platelet count × neutrophil count/lymphocyte count

SIRI = neutrophil count × monocyte count/lymphocyte count

PIV = platelet count × neutrophil count × monocyte count/lymphocyte count

All indices were calculated using laboratory measurements obtained at baseline before pacemaker implantation.

### 2.2. Study Endpoint and Follow-Up

The primary endpoint of the study was all-cause mortality during follow-up. Survival status and dates of death were obtained from institutional records and available electronic health records. Follow-up duration was calculated from the date of pacemaker implantation to the date of death or the last available clinical follow-up.

### 2.3. Statistical Analysis

Statistical analyses were performed using IBM SPSS Statistics, version 25. Categorical variables are presented as counts and column percentages, whereas continuous variables are presented as the mean ± standard deviation or median (interquartile range), according to their distribution. For categorical variables with missing data, percentages and between-group comparisons were based on the available observations for each variable. The numbers of available and missing observations for continuous baseline clinical and laboratory variables are summarized in Supplementary Table S1. The distributions of continuous variables were assessed using appropriate normality tests and visual inspection of histograms. Categorical variables were compared using the chi-square test or Fisher’s exact test, as appropriate. Normally distributed continuous variables were compared using the independent-sample *t* test, whereas non-normally distributed variables were compared using the Mann–Whitney U test. No imputation was performed. Descriptive and between-group analyses used available observations on a variable-by-variable basis, whereas the multivariable Cox model was based on complete cases for all included covariates (*n* = 237; 61 deaths).

Conventional receiver operating characteristic (ROC) analysis was performed to evaluate the discriminatory performance of inflammatory, nutritional, and conventional laboratory parameters for all-cause mortality. To permit direct descriptive comparison across the evaluated markers, ROC curves, AUCs, 95% confidence intervals, and *p* values were estimated in a common complete-case ROC sample with data available for all 12 markers (*n* = 222; 55 deaths). For variables inversely associated with mortality, AUC values were reported in the direction of increased risk. For exploratory purposes, the cohort-specific Youden cut-off and corresponding sensitivity, specificity, positive predictive value, negative predictive value, and Youden index were calculated using all available observations for each individual marker. Kaplan–Meier survival curves were generated according to the cohort-specific ROC-derived PNI cut-off and compared using the log-rank test.

Cox proportional hazards regression analysis was used to identify variables independently associated with all-cause mortality. Follow-up duration was entered as the time variable, and all-cause mortality status was entered as the event variable. Variables associated with mortality in univariable analyses or considered clinically relevant were included in the multivariable Cox regression model. To reduce the risk of overfitting given the observed number of mortality events, the multivariable model was parsimonious and limited to clinically relevant variables. The final model included PNI, hemoglobin, estimated glomerular filtration rate, age, pacemaker type, heart failure, and atrial fibrillation. Pacemaker type was retained as an adjustment covariate because device selection is influenced by rhythm status, comorbidity burden, frailty, and clinical judgment; the model was not designed to estimate a causal effect of pacing mode. The proportional hazards assumption was assessed by adding time-dependent interaction terms between each covariate and the natural logarithm of analysis time to the final Cox model. The global interaction test was not statistically significant (χ^2^ = 6.034, df = 7, *p* = 0.536), and none of the individual covariate-by-time interactions reached statistical significance, supporting the proportional hazards assumption. Hazard ratios with 95% confidence intervals and *p* values were reported. A two-sided *p* value < 0.05 was considered statistically significant.

Ethical approval details are provided in the Institutional Review Board Statement section.

## 3. Results

A total of 272 patients who underwent permanent pacemaker implantation for degenerative complete atrioventricular block were included. During a mean follow-up of 27.8 ± 16.6 months, all-cause mortality occurred in 69 patients (25.4%). Baseline categorical characteristics are presented in Table 1.

**Table 1:** Baseline categorical characteristics according to mortality status.

Variable	Survivors (*n* = 203)	Non-Survivors (*n* = 69)	*p* Value
Heart failure	39 (19.5%)	28 (41.8%)	<0.001 ^a^
Coronary artery disease	71 (35.5%)	31 (46.3%)	0.116 ^a^
Diabetes mellitus	67 (33.7%)	30 (44.8%)	0.102 ^a^
Hypertension	128 (64.0%)	47 (69.1%)	0.444 ^a^
Atrial fibrillation	53 (26.5%)	27 (40.3%)	0.033 ^a^
Sex			0.581 ^a^
Male	104 (51.2%)	38 (55.1%)	
Female	99 (48.8%)	31 (44.9%)	
Pacemaker type			<0.001 ^a^
Single-chamber	32 (15.8%)	27 (39.1%)	
Dual-chamber	171 (84.2%)	42 (60.9%)	

Data are presented as *n* (column percentage). Percentages were calculated using available cases within each mortality group. Valid denominators for survivors/non-survivors were 203/69 for sex and pacemaker type, 200/67 for heart failure, coronary artery disease, and atrial fibrillation, 199/67 for diabetes mellitus, and 200/68 for hypertension. Missing data were excluded on a variable-by-variable basis. ^a^ Pearson chi-square test; comparisons were based on raw counts for the presence and absence of each characteristic.

Heart failure was documented in 67 of 267 patients with available data (25.1%). Its prevalence was 19.5% among survivors and 41.8% among non-survivors (*p* < 0.001). Atrial fibrillation was also more frequent among non-survivors. Continuous laboratory parameters and inflammatory/nutritional indices are presented in Table 2.

**Table 2:** Comparison of continuous laboratory parameters and inflammatory/nutritional indices according to mortality status.

Variable	Survivors (*n* = 203)	Non-Survivors (*n* = 69)	*p* Value
Hemoglobin, g/dL	12.38 ± 1.93	11.03 ± 2.19	<0.001 ^a^
WBC, 10^9^/L	8.54 (3.03)	8.56 (6.12)	0.761 ^b^
Neutrophil count, 10^9^/L	5.50 (3.32)	5.76 (6.16)	0.526 ^b^
Lymphocyte count, 10^9^/L	1.75 (1.23)	1.36 (1.11)	0.001 ^b^
Monocyte count, 10^9^/L	0.67 (0.32)	0.85 (0.64)	0.165 ^b^
Platelet count, 10^9^/L	221.66 ± 65.07	220.98 ± 75.10	0.943 ^a^
AST, U/L	19.00 (11.00)	29.00 (28.75)	0.559 ^b^
ALT, U/L	16.00 (14.00)	15.50 (20.25)	0.056 ^b^
Creatinine, mg/dL	1.08 (0.60)	1.37 (1.13)	<0.001 ^b^
Total protein, g/L	66.00 (10.00)	64.50 (14.00)	0.133 ^b^
Albumin, g/L	40.00 (6.00)	34.50 (7.00)	<0.001 ^b^
Total cholesterol, mg/dL	161.00 (55.00)	143.00 (70.00)	0.390 ^b^
Triglycerides, mg/dL	112.00 (79.00)	107.50 (77.00)	0.492 ^b^
Sodium, mmol/L	139.00 (4.00)	138.00 (6.50)	0.094 ^b^
Potassium, mmol/L	4.45 ± 0.60	4.54 ± 0.59	0.283 ^a^
Age, years	72.00 (13.75)	83.50 (12.25)	<0.001 ^b^
BMI, kg/m^2^	28.65 (6.27)	25.71 (7.37)	0.125 ^b^
CRP, mg/L	5.00 (18.75)	16.40 (30.75)	0.016 ^b^
GFR, mL/min/1.73 m^2^	60.40 (40.68)	41.70 (32.10)	<0.001 ^b^
GNRI	111.99 ± 11.04	105.05 ± 13.71	<0.001 ^a^
NLR	2.77 (2.89)	3.53 (5.29)	0.012 ^b^
LMR	2.69 (1.31)	1.61 (1.82)	<0.001 ^b^
PIV	384.97 (430.20)	504.24 (1751.68)	0.068 ^b^
PNI	49.28 ± 7.14	44.00 ± 6.30	<0.001 ^a^
SII	590.76 (671.56)	727.02 (1468.36)	0.061 ^b^
SIRI	1.82 (1.68)	2.35 (6.18)	0.019 ^b^

Values are presented as the mean ± standard deviation for variables analyzed using the independent-sample *t* test and as the median (interquartile range) for variables analyzed using the Mann–Whitney U test. ^a^ Independent-sample *t* test; ^b^ Mann–Whitney U test. ALT, alanine aminotransferase; AST, aspartate aminotransferase; BMI, body mass index; CRP, C-reactive protein; GFR, glomerular filtration rate; GNRI, geriatric nutritional risk index; LMR, lymphocyte-to-monocyte ratio; NLR, neutrophil-to-lymphocyte ratio; PIV, pan-immune-inflammation value; PNI, prognostic nutritional index; SII, systemic immune-inflammation index; SIRI, systemic inflammation response index; WBC, white blood cell count.

Non-survivors were older and had lower hemoglobin, lymphocyte count, albumin, GFR, GNRI, LMR, and PNI values. In contrast, CRP, NLR, and SIRI were significantly higher among non-survivors. PIV and SII were numerically higher in non-survivors but did not reach statistical significance.

Receiver operating characteristic analysis was performed to evaluate the discriminatory performance of inflammatory, nutritional, and conventional laboratory parameters for all-cause mortality. The AUC estimates were derived from the common complete-case ROC sample (*n* = 222; 55 deaths), whereas cohort-specific cut-offs and operating characteristics were calculated using all available observations for each marker. Detailed results are presented in Table 3. PNI yielded the numerically highest AUC (0.720, 95% CI: 0.648–0.793; *p* < 0.001), corresponding to moderate discriminatory performance. At the exploratory, cohort-specific ROC-derived cut-off of ≤48.90, PNI yielded a sensitivity of 82.8%, specificity of 53.4%, PPV of 39.0%, NPV of 89.6%, and Youden index of 0.362. GNRI showed fair discrimination (AUC: 0.677, 95% CI: 0.589–0.766; *p* < 0.001), with lower sensitivity (57.6%) but higher specificity (78.1%) than PNI. Albumin, GFR, hemoglobin, age, LMR, and lymphocyte count also demonstrated significant discriminatory performance. In contrast, SII, SIRI, NLR, and PIV had lower AUC values and were not statistically significant. Because formal pairwise comparisons of AUCs were not performed, the observed differences should be interpreted descriptively.

**Figure 1:**
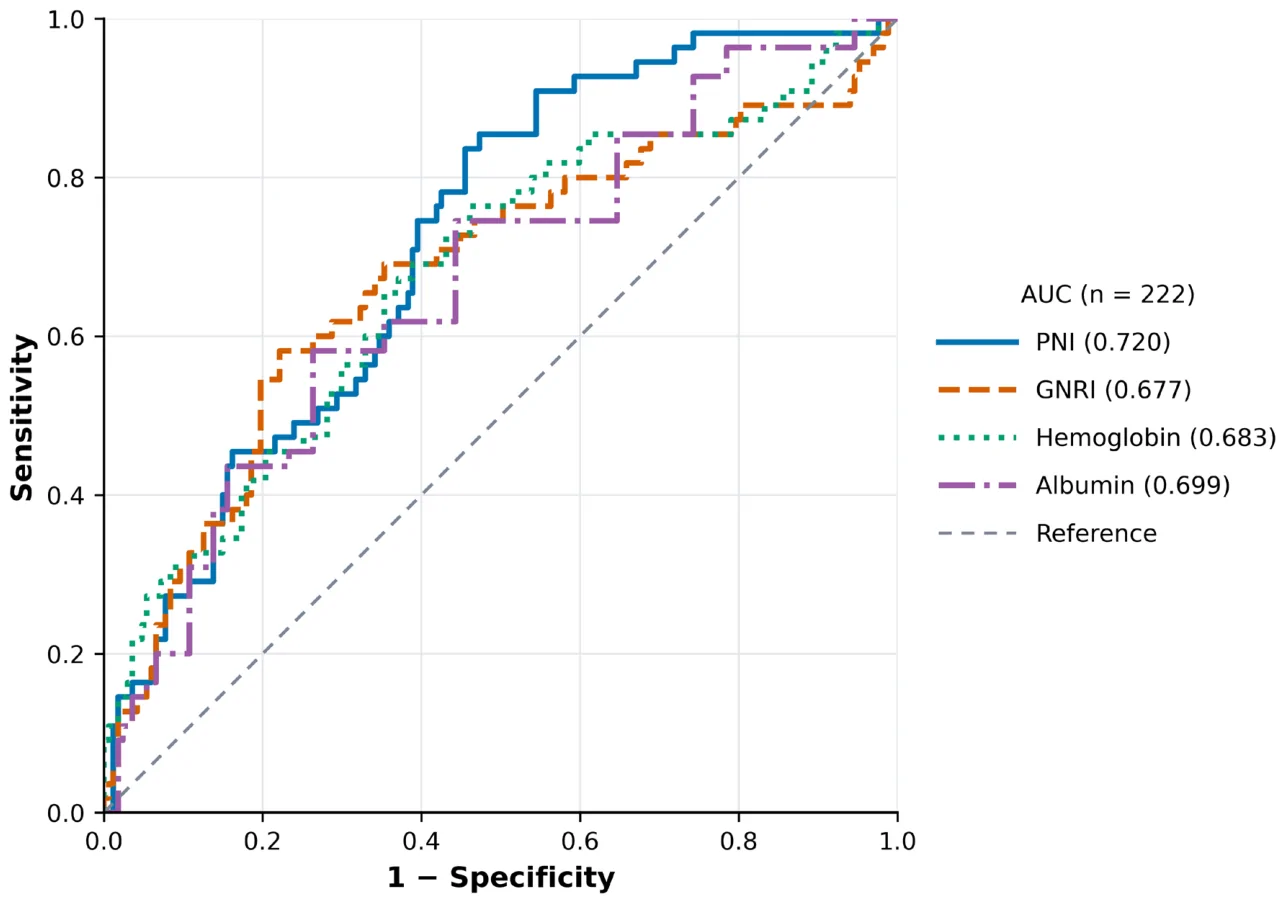
Receiver operating characteristic curves for prediction of all-cause mortality. Abbreviations: ROC, receiver operating characteristic; PNI, prognostic nutritional index; GNRI, geriatric nutritional risk index. Curves and AUCs were derived from the common complete-case ROC sample (*n* = 222; 55 deaths).

In ROC analysis, the prognostic nutritional index (PNI) had the numerically highest AUC among the evaluated markers. The cohort-specific ROC-derived PNI cut-off selected using the Youden index was ≤48.90, with a sensitivity of 82.8% and a specificity of 53.4%. Given its modest specificity and the use of conventional ROC analysis in a time-to-event data set, this value should be regarded as exploratory and cohort-specific rather than as a definitive clinical threshold. It may support adjunctive risk stratification but requires external validation before clinical application (Figure 1).

**Figure 2:**
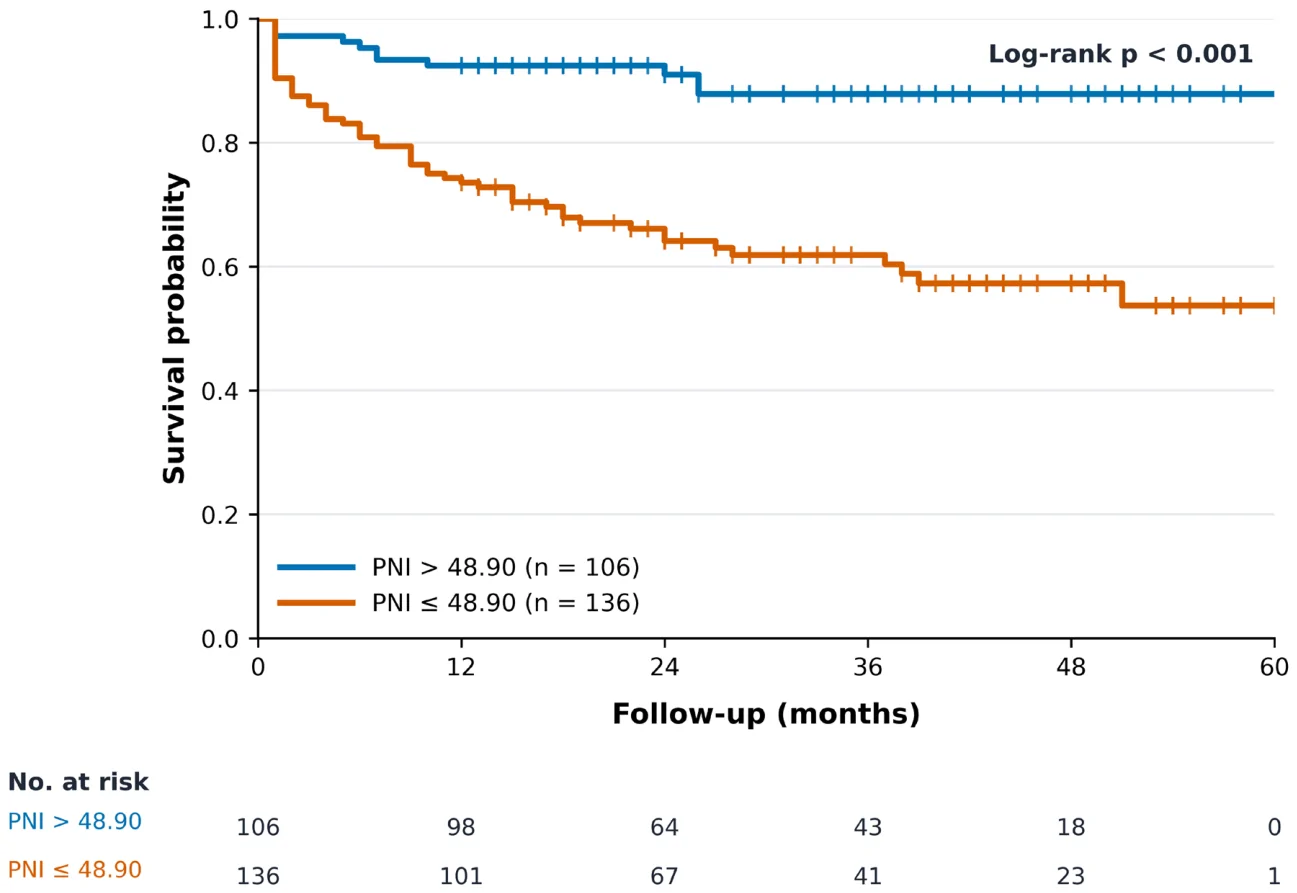
Kaplan–Meier survival curves according to PNI cut-off value. Abbreviations: PNI, prognostic nutritional index. Multivariable Cox regression findings are summarized in Table 4.

Kaplan–Meier analysis demonstrated significantly lower cumulative survival among patients with a PNI ≤ 48.90 than among those with a PNI > 48.90. The survival curves differed significantly according to the log-rank test (χ^2^ = 23.435, df = 1, *p* < 0.001), indicating that a lower PNI was associated with a higher mortality risk during follow-up (Figure 2).

**Table 4:** Multivariable Cox proportional hazards regression analysis for all-cause mortality.

Variable	HR	95% CI	*p* Value
PNI	0.945	0.909–0.982	0.004
Single-chamber pacemaker vs. dual-chamber pacemaker	2.139	1.201–3.808	0.010
Hemoglobin, g/dL	0.882	0.771–1.009	0.067
GFR, mL/min/1.73 m^2^	0.992	0.981–1.002	0.125
Age, years	1.016	0.990–1.043	0.232
Heart failure	1.258	0.691–2.289	0.453
Atrial fibrillation	0.907	0.500–1.642	0.746

CI, confidence interval; GFR, estimated glomerular filtration rate; HR, hazard ratio; PNI, prognostic nutritional index. Continuous predictors are expressed per 1-unit increase. The multivariable model included 237 complete cases and 61 mortality events.

In multivariable Cox proportional hazards regression analysis, PNI remained independently associated with all-cause mortality after adjustment for hemoglobin, renal function, age, pacemaker type, heart failure, and atrial fibrillation. Each 1-point increase in PNI was associated with a 5.5% lower mortality hazard (HR: 0.945, 95% CI: 0.909–0.982; *p* = 0.004). Pacemaker type was retained in the model only as an adjustment covariate, and no causal comparison of pacing modes was intended.

## 4. Discussion

In this retrospective study of patients undergoing permanent pacemaker implantation for degenerative complete atrioventricular block, the principal finding was that PNI had the numerically highest discriminatory performance among the evaluated inflammatory and nutritional indices and remained independently associated with long-term all-cause mortality after adjustment for hemoglobin, renal function, age, pacemaker type, heart failure, and atrial fibrillation. PNI also yielded a numerically higher AUC than either serum albumin or lymphocyte count alone, suggesting that an integrated immune-nutritional index may capture prognostically relevant information beyond either component considered separately.

The present study focused on a homogeneous population with isolated complete atrioventricular block attributed to degenerative conduction system disease. This focus is clinically relevant because complete atrioventricular block may arise from several secondary or iatrogenic conditions, whereas degenerative conduction system disease generally occurs in older and more vulnerable patients. Contemporary studies of mortality and frailty among pacemaker recipients have identified advanced age, diabetes mellitus, reduced left ventricular systolic function, anemia, renal dysfunction, structural heart disease, and frailty as adverse prognostic factors [2,3,5,6]. However, these studies generally included heterogeneous pacing indications or device populations. Restricting the study population to degenerative complete atrioventricular block enabled a focused evaluation of systemic vulnerability, nutritional status, and inflammation in a clinically uniform subgroup.

PNI was originally developed as a nutritional and immunological index using serum albumin and lymphocyte count [7]. Serum albumin reflects protein-calorie reserve, hepatic synthetic function, inflammation-related capillary leakage, and overall disease burden, whereas lymphocyte count reflects immune competence and may decrease in chronic inflammation, malnutrition, and physiological stress. Therefore, PNI may capture the interaction between nutritional depletion and immune-inflammatory dysfunction more comprehensively than albumin or lymphocyte count alone. Importantly, the absence of a documented diagnosis of malnutrition does not exclude subclinical immune-nutritional impairment. Our cohort was predominantly older, with median ages of 72.0 years among survivors and 83.5 years among non-survivors, making assessment of physiological and nutritional reserve clinically relevant. Contemporary cardiovascular studies have also associated lower PNI with adverse long-term outcomes [17,20], and poor nutritional status has been linked to arrhythmic events [19]. Furthermore, a recent study of 927 patients with cardiac pacemakers showed that both low baseline PNI and deterioration in PNI during follow-up were associated with heart failure hospitalization and all-cause mortality [9], supporting the applicability of PNI beyond its original surgical oncology setting. In our ROC analysis, PNI had a numerically higher AUC than albumin and lymphocyte count. Moreover, although albumin, lymphocyte count, GNRI, LMR, and several inflammatory markers differed between survivors and non-survivors, only PNI remained independently associated with mortality in the multivariable model.

The prognostic importance of albumin in cardiovascular disease has been increasingly recognized. Among patients with permanent pacemakers, Hayıroğlu et al. reported an association between serum albumin and long-term mortality in recipients of dual-chamber devices [4]. In broader cardiovascular populations, low albumin has also been linked to adverse outcomes and may represent a global marker of nutritional impairment, chronic inflammation, frailty, and reduced physiological reserve [23]. Our findings extend these observations: PNI, which integrates albumin and lymphocyte count, yielded a numerically higher AUC than albumin alone for mortality prediction in patients with degenerative complete atrioventricular block. This finding suggests that the prognostic signal in this population may reflect broader immune-nutritional impairment rather than hypoalbuminemia alone.

Other inflammatory and nutritional indices also provided relevant information. GNRI, LMR, NLR, and SIRI differed significantly between survivors and non-survivors, whereas SII and PIV were numerically higher among non-survivors but were not statistically significant. GNRI was originally developed to assess nutrition-related risk in older medical patients [8] and has subsequently demonstrated prognostic value in older cardiovascular populations [10,16]. Contemporary data also support the association of NLR with all-cause and cardiovascular mortality in cardiovascular populations [18]. These observations support the relevance of such indices to our predominantly older pacemaker cohort, even though patients were not selected on the basis of overt malnutrition. PNI yielded a numerically higher AUC than GNRI (0.720 vs. 0.677), corresponding to moderate and fair discrimination, respectively. However, the confidence intervals overlapped, and no formal pairwise AUC comparison was performed; therefore, these findings do not establish statistically superior discrimination by PNI. The performance profiles of the two indices were complementary: PNI had higher sensitivity than GNRI (82.8% vs. 57.6%) but lower specificity (53.4% vs. 78.1%). Thus, PNI identified a larger proportion of patients who died during follow-up, whereas GNRI provided more specific classification. The numerically higher discrimination of PNI may partly reflect the inclusion of lymphocyte count, which captures immune-inflammatory dysfunction in addition to serum albumin. By contrast, GNRI incorporates body weight relative to ideal weight. BMI did not differ significantly between survivors and non-survivors in our cohort (*p* = 0.125), which may have limited the incremental contribution of the weight-based component. Accordingly, both indices should be regarded as complementary adjunctive risk markers rather than standalone prognostic tools.

Pacemaker type was retained only as an adjustment covariate. Because device selection is closely related to rhythm status, frailty, comorbidity burden, and physician judgment, the observed association with mortality should be considered secondary and hypothesis-generating rather than evidence of a causal effect of pacing mode [24]. Future studies specifically designed to evaluate pacing mode should prospectively record the clinical rationale for device selection and detailed measures of rhythm status, frailty, functional capacity, and comorbidity burden. Propensity-score matching or weighting and other causal-inference approaches may reduce confounding by indication; nevertheless, residual confounding may persist in non-randomized comparisons.

The principal clinical implication of this study is that PNI may serve as a readily available adjunctive marker for risk stratification in patients undergoing permanent pacemaker implantation for degenerative complete atrioventricular block. Because PNI is calculated from routine preprocedural laboratory parameters and requires no additional testing, it may help identify patients who warrant closer clinical follow-up, formal assessment of frailty and nutritional status, and optimization of modifiable systemic risk factors. Whether interventions targeting nutritional status or systemic inflammation improve outcomes in this population remains uncertain. Future pilot and randomized feasibility studies could evaluate structured nutritional optimization and frailty-directed physical rehabilitation in patients with low PNI. Because pacemaker implantation for complete atrioventricular block is often urgent and generally cannot be delayed for preprocedural rehabilitation, interventions initiated immediately after implantation may represent the most clinically feasible strategy.

The mortality observed in our cohort may also reflect the specific characteristics of the study population. The Czech national registry reported higher long-term survival among patients undergoing pacemaker implantation [25]. In contrast, our study exclusively included patients with degenerative complete atrioventricular block and excluded secondary, iatrogenic, and congenital causes, thereby defining a more clinically uniform but potentially more vulnerable group. This strict selection enhanced the internal consistency of the cohort and supports the relevance of immune-nutritional risk stratification in this population.

### Limitations

This study has several limitations. First, its retrospective, single-center design may limit the generalizability of the findings and precludes causal inference. Second, although PNI was independently associated with mortality, it should be regarded as an indirect marker of immune-nutritional vulnerability rather than a direct measure of frailty or nutritional status. Frailty, nutritional status, functional capacity, and sarcopenia were not systematically assessed using validated instruments such as gait speed, handgrip strength, the Clinical Frailty Scale, or the Mini Nutritional Assessment. Consequently, the incremental prognostic value of PNI beyond formal frailty and nutritional assessments could not be determined. Third, the inflammatory and nutritional indices were calculated only from baseline laboratory values. Serial measurements at discharge and during follow-up were unavailable; therefore, longitudinal changes in PNI, albumin, lymphocyte count, and inflammatory markers, as well as whether these indices change after pacemaker implantation, could not be evaluated. Prospective studies incorporating repeated measurements are needed to determine whether dynamic trajectories provide prognostic information beyond baseline values. Fourth, post-implantation pharmacotherapy—including guideline-directed heart failure therapy, anticoagulant, antiplatelet, and lipid-lowering treatment—together with medication dose, adherence, and longitudinal treatment changes, was not systematically available. The potential influence of medical therapy on mortality could therefore not be evaluated, and residual confounding cannot be excluded. Fifth, pacemaker type was not randomly assigned; therefore, its association with mortality may have been influenced by confounding by indication, baseline clinical vulnerability, and device-selection patterns. Although pacemaker type was included as an adjustment covariate, conventional multivariable adjustment cannot eliminate unmeasured confounding. More granular covariate collection and propensity-score methods may reduce, but not eliminate, this bias in future observational studies. Sixth, detailed echocardiographic parameters, including left ventricular ejection fraction, chamber dimensions, and valvular variables, were not systematically available and could not be included in the multivariable analysis. Seventh, sufficiently detailed data on pacing burden, device programming, and cause-specific mortality were unavailable. Eighth, conventional ROC analysis does not fully account for time-to-event information; therefore, the ROC-derived cut-off and discriminatory findings should be considered cohort-specific and exploratory. Finally, external validation in larger prospective cohorts is required to confirm the prognostic value of PNI in this patient population.

## 5. Conclusions

Among the evaluated inflammatory and nutritional indices, PNI demonstrated the numerically highest discriminatory performance and remained independently associated with long-term all-cause mortality in patients undergoing permanent pacemaker implantation for degenerative complete atrioventricular block. These findings support PNI as a readily available adjunct to clinical risk stratification, although its incremental clinical value and cohort-specific cut-off require external validation. Prospective multicenter studies should validate the prognostic role of PNI, and pilot randomized feasibility trials should determine whether early post-implantation nutritional optimization or frailty-directed rehabilitation improves outcomes in this population.

## Figures and Tables

**Table 3:** Receiver operating characteristic curve analysis and diagnostic performance metrics for mortality prediction.

Variable	AUC (95% CI)	*p* Value	Cut-Off	Sensitivity (%)	Specificity (%)	PPV (%)	NPV (%)	Youden Index
PNI	0.720 (0.648–0.793)	<0.001	≤48.90	82.8	53.4	39.0	89.6	0.362
Albumin, g/L	0.699 (0.620–0.778)	<0.001	≤37.50	57.8	73.7	44.0	83.0	0.315
GFR, mL/min/1.73 m^2^	0.686 (0.607–0.765)	<0.001	≤73.15	79.4	47.5	33.8	87.3	0.269
Hemoglobin, g/dL	0.683 (0.599–0.768)	<0.001	≤12.15	69.6	56.4	35.3	84.4	0.260
GNRI	0.677 (0.589–0.766)	<0.001	≤104.27	57.6	78.1	47.9	84.1	0.357
Age, years	0.668 (0.588–0.748)	<0.001	≥74.50	71.0	54.7	34.8	84.7	0.257
LMR	0.655 (0.568–0.743)	0.001	≤2.22	53.0	74.1	40.2	82.8	0.271
Lymphocyte count, 10^9^/L	0.637 (0.553–0.721)	0.002	≤1.79	72.5	55.4	35.7	85.5	0.279
SIRI	0.580 (0.488–0.671)	0.077	≥1.57	71.2	46.3	30.3	83.0	0.175
NLR	0.574 (0.486–0.663)	0.099	≥2.48	71.0	44.1	30.2	81.7	0.151
PIV	0.558 (0.463–0.653)	0.197	≥666.88	39.4	76.6	35.6	79.4	0.160
SII	0.552 (0.459–0.645)	0.250	≥1616.57	26.1	91.1	50.0	78.3	0.172

Abbreviations: AUC, area under the curve; CI, confidence interval; GFR, estimated glomerular filtration rate; GNRI, geriatric nutritional risk index; LMR, lymphocyte-to-monocyte ratio; NLR, neutrophil-to-lymphocyte ratio; NPV, negative predictive value; PIV, pan-immune-inflammation value; PNI, prognostic nutritional index; PPV, positive predictive value; SII, systemic immune-inflammation index; SIRI, systemic inflammation response index. AUCs and corresponding 95% CIs were estimated in the common complete-case ROC sample (*n* = 222; 55 deaths) to allow direct descriptive comparison across markers. For variables inversely associated with mortality, lower values were considered to indicate increased risk. Cut-offs, sensitivity, specificity, PPV, NPV, and Youden indices were calculated using all available observations for each individual marker.

## Data Availability

The data presented in this study are available from the corresponding author upon reasonable request due to privacy and ethical restrictions.

## Supplementary Materials

The following supporting information can be downloaded at: https://www.mdpi.com/article/10.3390/medicina62091741/s1, Supplementary Table S1. Availability of continuous baseline clinical and laboratory variables.
