# Supplementary material for: Prognostic Value of Inflammatory and Nutritional Indices in Patients Undergoing Permanent Pacemaker Implantation for Degenerative Complete Atrioventricular Block
Source: Medicina (Kaunas). 2026 Sep 10;62(9):1741. doi: 10.3390/medicina62091741 (PMC13609748; doi:10.3390/medicina62091741)
Supplement: Supplementary file 1 [file medicina-62-01741-s001.zip › medicina-4440361-supplementary.pdf]

Supplementary Materials

Prognostic Value of Inflammatory and Nutritional Indices in Patients Undergoing Permanent Pacemaker Implantation for Degenerative Complete Atrioventricular Block

Supplementary Table S1. Availability of continuous baseline clinical and laboratory variables

Variable	Available observations, n	Missing observations, n	Completeness, %
Hemoglobin	271	1	99.6
Body mass index	257	15	94.5
White blood cell count	271	1	99.6
Neutrophil count	271	1	99.6
Platelet count	271	1	99.6
Lymphocyte count	271	1	99.6
Monocyte count	267	5	98.2
C-reactive protein	256	16	94.1
Estimated glomerular filtration rate	270	2	99.3
Creatinine	271	1	99.6
Urea	271	1	99.6
Aspartate aminotransferase	262	10	96.3
Alanine aminotransferase	263	9	96.7
Sodium	271	1	99.6
Potassium	269	3	98.9
Triglycerides	222	50	81.6
Low-density lipoprotein cholesterol	158	114	58.1
High-density lipoprotein cholesterol	159	113	58.5
Total cholesterol	222	50	81.6
Total protein	166	106	61.0
Albumin	243	29	89.3
Prognostic nutritional index (PNI)	242	30	89.0
Geriatric nutritional risk index (GNRI)	228	44	83.8
Neutrophil-to-lymphocyte ratio (NLR)	271	1	99.6
Lymphocyte-to-monocyte ratio (LMR)	267	5	98.2
Systemic immune-inflammation index (SII)	271	1	99.6
Systemic inflammation response index (SIRI)	267	5	98.2
Pan-immune-inflammation value (PIV)	267	5	98.2

Note: Total cohort, N = 272. Only variables with at least one missing observation are shown. No imputation was performed; descriptive and between-group analyses used the available observations for each variable.
